# Critical Review on the Development and Evolution of Beat Perception

**DOI:** 10.1111/nyas.70386

**Published:** 2026-09-10

**Authors:** Gábor Háden, Henkjan Honing

**Affiliations:** ^1^ Institute of Cognitive Neuroscience and Psychology HUN‐REN Research Centre for Natural Sciences Budapest Hungary; ^2^ Music Cognition Group, Institute for Logic, Language, and Computation, Amsterdam Brain and Cognition University of Amsterdam Amsterdam the Netherlands

**Keywords:** beat perception and synchronization, comparative cognition, musicality, ontogeny, phylogeny

## Abstract

Beat perception—the ability to extract a regular pulse from rhythmic sequences—is a foundational component of human musicality and a key mechanism supporting synchronization, dance, and collective music‐making. Research across neuroscience, developmental psychology, and comparative cognition demonstrates that beat processing exhibits four defining characteristics: it is near‐universal across human cultures, emerges spontaneously in early development, engages domain‐specific predictive timing mechanisms, and appears rare across species. Neurophysiological evidence indicates that newborns respond to rhythmic regularities using predictive neural processes that cannot be explained by simple interval timing or statistical learning alone. Across infancy and childhood, these early predispositions are progressively refined through auditory experience, motor development, and musical enculturation. Comparative studies show—although the evidence is still sparse—that several species (including humans) share this ability, but also that nonhuman primates, while able to match the tempo of an auditory sequence, typically do not show robust beat synchronization. Together, findings from phylogeny and ontogeny suggest that beat perception reflects an early‐emerging, biologically prepared capacity that is further shaped by experience. Understanding its developmental and evolutionary bases offers crucial insight into the origins of human musicality and the neural architecture supporting temporal prediction.

## Graphical abstract

Beat perception—extracting a regular pulse from rhythm—supports human musicality, synchronization, and dance. Evidence suggests it is widespread across cultures, emerges at or before birth, and is rare across species. Newborn neurophysiology indicates beat‐sensitive prediction beyond simple interval timing or statistical learning, which experience and motor development then refine. Nonhuman primates can match the tempo of auditory sequences but typically do not show robust predictive beat synchronization, though a few other species (including humans) do.

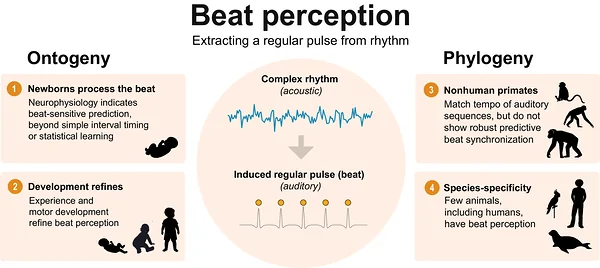

## 1 Introduction

Beat perception can be defined as the cognitive ability to extract a regular pulse from a varying rhythmic pattern [1]. It is often described as a trivial skill because it enables common behaviors such as clapping along with a song, tapping a foot to music, or dancing in synchrony with others [2]. Recent research has shown that this ability is fundamental to human musicality [3, 4]. Beat perception facilitates coordination, dance, and ensemble performance, suggesting that it is not only central to musical engagement but may also have played a decisive role in the evolutionary origins of music [5, 6]. In the following, beat perception (or more precisely, *beat processing*; see below) will be considered from four complementary perspectives: its apparent universality, spontaneous development, domain‐specificity, and species‐specificity. This is followed by a critical review of current literature reviewing both its phylogenetic and ontogenetic foundations.

### 1.1 Characteristics of Beat Processing

#### 1.1.1 Omnipresent

The beat (or regular pulse) is a near‐universal feature of human musical culture [7]. Despite substantial cross‐cultural variation in rhythmic preferences and performance practices [8], virtually all known musical traditions exhibit temporal regularities that afford the perception of a pulse. The ability to entrain to such a beat underpins collective music‐making, allowing groups of individuals to synchronize their movements and sound production. Thus, beat perception can be seen as a candidate for a universal cognitive trait—a shared aspect of human nature that transcends cultural diversity and facilitates social cohesion [2].

#### 1.1.2 Spontaneously Developing

The ability and motivation of humans—and, as far as we currently know, just a few other species (see below)—to move in time with a musical beat appears to emerge without explicit instruction, while still depending on species‐typical developmental inputs, ecological regularities, and experience [9, 10]. Hierarchical beat perception develops throughout adolescence [11]; however, recent evidence suggests that beat perception may be present from birth. Studies using mismatch negativity (MMN) paradigms report that newborns, including 2‐ to 3‐day‐old infants, already show neural responses consistent with beat perception [11, 12, 13]. These findings support the view that beat perception develops spontaneously, though the role of reward and the extent of learning‐dependent refinement remain under debate.

#### 1.1.3 Domain‐Specific

Another line of inquiry concerns whether beat perception reflects a domain‐general timing mechanism or a domain‐specific adaptation for music [14, 15]. Humans can perceive absolute temporal intervals, learn statistical regularities in auditory sequences, and detect transitional probabilities [16]. However, beat‐based timing appears to be distinct from these processes [13, 17]. Unlike interval timing, beat perception involves projecting temporal regularities forward in time, enabling predictive synchronization of perception and action. Neural evidence points to entrainment of low‐frequency oscillations in auditory and motor areas as a specialized mechanism supporting beat perception [18, 19, 20]. Thus, beat perception can be argued to constitute a domain‐specific process, tailored to the auditory system and the demands of rhythmic engagement with music. Note that predictive sensorimotor timing is not unique to audition: visuomotor systems clearly support spatiotemporal anticipation even in silent scenarios. Nevertheless, *beat‐based* timing shows a reliable auditory advantage (with tactile typically intermediate and visual weakest) in both behavior and neural measures [19, 21].

#### 1.1.4 Species‐Specific (i.e., Rare Across Species)

Comparative research suggests that beat‐based synchronization may be limited to a small subset of species. However, because the comparative evidence remains sparse, the current picture is provisional: humans (from early in development) readily show spontaneous auditory–motor entrainment to a beat, whereas clear evidence for predictive beat‐based synchronization in nonhuman species is scarce, in large part because so few species have been examined. Accordingly, we do not claim that beat processing is “typically human” by definition. Some bird species (e.g., parrots, cockatoos) have demonstrated entrainment behaviors [22], and occasional individual animals (of other species) have shown partial synchronization [23, 24, 25]. However, spontaneous auditory−motor coupling to rhythm has not been observed across all primates, despite their close phylogenetic proximity to humans [21]. This suggests that beat perception may be a cognitive specialization of just a few species, potentially linked to the coevolution of music, dance, and social bonding [26].

Taken together, *beat processing* appears to be a universal, spontaneously developing, domain‐specific, and species‐specific skill. Its role in enabling predictive synchronization and collective coordination strongly supports the hypothesis that beat perception and synchronization was decisive in the origins of music and musicality [27]. Understanding its cognitive, developmental, and biological foundations not only deepens our knowledge of human music perception but also sheds light on the broader question of how uniquely human music capacities evolved [5, 6, 28, 29].

### 1.2 Conceptual and Methodological Challenges

Beat perception must be carefully dissociated from the detection of statistical regularities in event sequences, such as transitional or conditional probabilities, which have been extensively studied in language [30] and tonal stimuli [31]. Statistical learning, defined as the extraction of such regularities without explicit feedback or awareness after exposure [32], is considered a domain‐general mechanism evident across multiple modalities, including audition [31], music [33], and vision [34]. It is most prominently associated with language and appears functional at birth [35].

In the context of rhythm processing in infants, it is, therefore, essential to distinguish beat perception from interval timing and statistical learning. To demonstrate beat perception, rhythmic stimuli must contain sufficient acoustic or temporal variation to separate it from simple interval‐based processing. At the same time, acoustic variation (e.g., differences in pitch or intensity) may facilitate beat detection, particularly in novices [36], but also risks confounding results if responses attributed to beat perception are instead driven by acoustic or sequential properties. Establishing genuine evidence for beat perception thus requires experimental designs that rigorously control for these alternative explanations, for example, by selectively making the beat‐based interpretation harder or impossible with timing changes while keeping sequential statistical properties constant [37]. Some empirical examples are provided further below. Another caveat to consider is that in research involving participants that are not adult humans—such as preverbal infants, sleeping newborns, or nonhuman animals—a central challenge lies in distinguishing neural processing of sensory input from conscious perception of the environment. Brains may automatically register and transform incoming stimuli, yet such processing does not necessarily imply subjective awareness or explicit representation. (N.B. We will use the terms *beat processing* and *beat perception* interchangeably.)

A closely related problem is differentiating the capacity to process information and the ability to express such capacity through behavioral responses. Although modern methods, including direct neural measurements and behavioral paradigms, are designed to probe these questions, the distinction between neural processing and conscious perception, and between competence and behavioral expression, remains unresolved, representing a core conceptual and empirical challenge in developmental and comparative studies of rhythm, music, and cognition.

### 1.3 Phylogeny and Ontogeny of Beat Processing

In the following, we review recent literature on the phylogeny (evolutionary origins) and ontogeny (development) of beat perception, as a core component of the human capacity for music [2]. For this, comparative and developmental approaches are particularly informative. On the one hand, studies of human newborns and infants provide insights into the biological predispositions underlying beat processing and its early developmental trajectory. On the other hand, comparative research allows us to situate humans in a broader context, examining both closely related primates, who share substantial genetic and neurobiological similarities with humans, and more distantly related species, such as birds, whose neural architectures differ considerably yet sometimes exhibit rhythmic synchronization [38]. By combining evidence from ontogeny and phylogeny, this framework seeks to clarify whether beat perception is a unique human specialization, a shared trait with deep evolutionary roots, or a convergent adaptation in species with very different evolutionary histories [19, 39, 40, 41].

## 2 Phylogeny

Whether beat perception is uniquely human remains debated [40, 42, 43, 44]. Behavioral evidence for beat perception in nonhuman species has primarily relied on demonstrations of motor entrainment to a musical beat [37]. While such synchronization has been observed in a handful of species, notably certain birds and sea lions [38], the absence of overt motor behavior cannot be taken as conclusive evidence for the absence of beat perception. Some animals may sense a beat but lack the motor capacities to express it in synchrony. Moreover, behavioral methods confound perception and action, making it difficult to isolate underlying mechanisms [45]. But note that very few nonhuman species have been examined to date, limiting the strength of any comparative conclusions [43].

Electrophysiological approaches offer a promising alternative. Event‐related potentials (ERPs) and related measures can probe auditory processing without requiring overt motor responses. While early animal studies relied on invasive electrodes [46, 47], noninvasive scalp recordings have increasingly been used in nonhuman primates, allowing direct comparisons with human data [48, 49]. These methods have informed animal models of human auditory processing, elucidated the neural generators of ERP components, and revealed cross‐species similarities and differences in rhythm perception [50, 51].

MMN and its analogues in animals (often termed the mismatch response, MMR) are particularly relevant [52]. MMRs have been demonstrated across multiple species—including rodents, cats, macaques, and chimpanzees—using deviations in frequency, amplitude, or timing [49, 53]. These findings suggest that expectancy‐violation responses are not unique to humans. However, sensitivity to temporal regularity should not be equated with beat perception. Although nonhuman primates can detect isochrony and temporal deviations in rhythmic sequences, current evidence suggests that their ability to infer and maintain an induced beat is more limited [45]. This aligns with the gradual audiomotor evolution (GAE) hypothesis, which proposes that spontaneous beat perception and synchronization [20] evolved progressively within the primate lineage, reaching its most flexible and developed form in humans [54, 55].

An alternative account, the vocal learning and rhythmic synchronization (VLRS) hypothesis, links beat synchronization to neural circuits for complex vocal learning [38, 56]. Support comes from vocal‐learning birds such as parrots and cockatoos, which can synchronize to music across tempi [57, 58]. Yet, counterexamples challenge the strict association with vocal learning, such as a California sea lion capable of tempo‐flexible entrainment despite not being a vocal learner [24, 44, 59].

**FIGURE 1:**
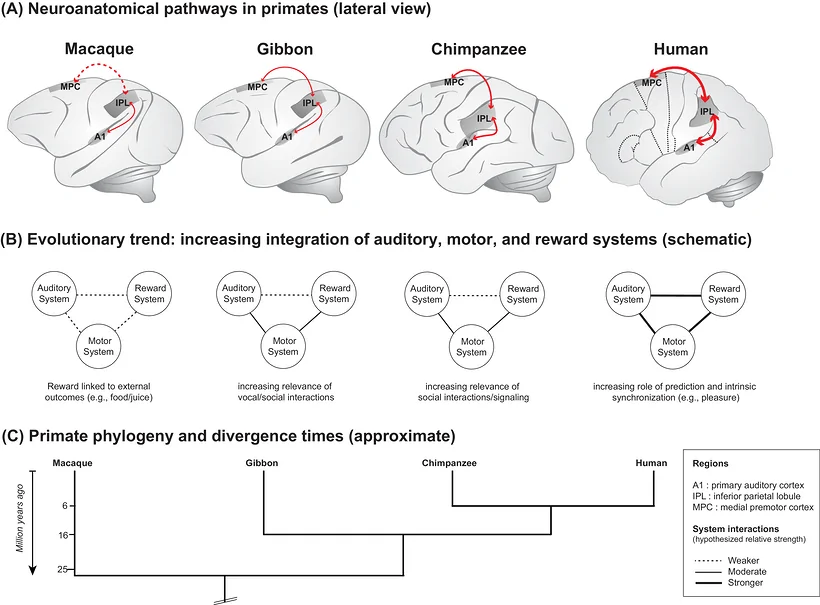
Gradual audio‐motor evolution with reward (GAER) hypothesis. GAER proposes that beat perception and synchronization emerged gradually within the primate lineage through the progressive strengthening and integration of auditory, motor, predictive‐timing, and reward‐related systems. The hypothesis distinguishes component abilities—including isochrony detection, internal maintenance of tempo, and tempo adaptation—from full beat‐based synchronization, which additionally requires predictive and flexible control of both period and phase. Nonhuman primates can approach human performance in several component processes, particularly isochrony detection and tempo matching, but generally show lower precision, greater phase variability, stronger dependence on training and extrinsic reward, and less flexible generalization than humans. Extending the original GAE account, GAER proposes that the progressive integration of auditory–motor circuits, cortico‐basal‐ganglia–thalamocortical timing networks, and reward‐related mechanisms supported the learning, motivation, temporal prediction, and stabilization necessary for spontaneous and flexible beat perception and synchronization (BPS) in humans. (A) Principal auditory–motor pathways in the primate brain. MPC, medial premotor cortex; IPL, inferior parietal lobule; A1, primary auditory cortex. Reward circuitry is not shown. Line thickness represents the hypothesized relative strength of functional and anatomical connectivity. (B) Hypothesized interactions among auditory, motor, and reward systems. Bidirectional connections support auditory prediction, motor planning, reinforcement learning, and rhythmic synchronization. Line thickness represents the hypothesized relative strength of connectivity (derived from [55] and [67]). (C) Primate phylogeny. Schematic phylogenetic relationships among the primate species. Evidence is drawn from comparative studies in rhesus macaques (*Macaca mulatta*) [45, 64], gibbons (*Hylobates* spp.) [154], chimpanzees (*Pan troglodytes*) [23, 155, 156, 157], and humans (*Homo sapiens*) [37]. Panel A is adapted from Ref. [39].

More recently, the “four‐components” (4Cs) framework has proposed that beat perception and synchronization should be viewed as a continuum reflecting the coordinated operation of four functional processes: auditory pattern detection, prediction, auditory–motor feedback, and reward‐based reinforcement learning [25, 60]. Unlike the VLRS and GAE hypotheses, however, the 4Cs framework is primarily a functional decomposition of beat perception and synchronization rather than an evolutionary or mechanistic hypothesis explaining how these capacities arose. In contrast, GAE proposes that these functional capacities emerged through the gradual strengthening and integration of conserved auditory–motor circuits across the primate lineage (with the updated gradual audiomotor evolution with reward [GAER] hypothesis explicitly addressing the role of the reward system; see next section and Figure 1), whereas VLRS attributes their evolution primarily to adaptations associated with complex vocal learning. Yet, both hypotheses highlight the importance of sensorimotor networks in the brain, including dorsal pathways. See Proksch et al. for an elaborate discussion [60].

Recent comparative evidence provides an important test of these competing interpretations. A study using overt behavior, while carefully controlling reward, demonstrated that macaques can adapt their tapping period to the tempo of human music (i.e., “tempo matching”), but do not show the stable phase alignment characteristic of human synchronization [25]. Although the authors state that the “monkeys synchronize” (see caption of Figure 1 in [25]), neither macaque shows the expected tapping phase (around 0°), nor do they show clear evidence of successful phase shifting (in the 180° condition). The claim that “macaques can synchronize to a subjective beat in real music” is, therefore, not supported by the reported data [61].

While macaques can adapt their tapping tempo to human music after extensive reward‐based training, the underlying process likely differs substantially from that observed in humans. Unlike human participants, who required no training, the macaques depended on prolonged reinforcement and displayed behavioral signs of effort and frustration [61]. This implies that synchronization to music is not intrinsically rewarding for them, in contrast to humans, for whom audiomotor synchronization often engages the reward system and supports culturally central practices such as music, dance, and speech. Humans might well differ from macaques in the functional connectivity between auditory, motor, and reward circuits, with reward‐related structures mediating stronger audiomotor integration [62]. Quantitatively, macaques matched human performance across tempi but displayed much greater phase variability in tapping [25], consistent with earlier studies [63, 64, 65] and with the GAE hypothesis, which we extend below into the GAER hypothesis (see next section).

Taken together, these studies suggest that sensitivity to isochrony is widespread across species, while genuine beat perception—the capacity for predictive, tempo‐flexible synchronization—remains rare [38]. It appears most fully developed in humans, but limited in other primates, present in some vocal‐learning birds, and occasionally observed in unexpected species [66].

### 2.1 The GAER Hypothesis: Extending the GAE Model

Building on the recent findings reviewed above, the GAER hypothesis extends the original GAE model by explicitly incorporating reward processing into the evolution of auditory–motor timing. GAER proposes that beat perception and synchronization emerged gradually within the primate lineage through the progressive strengthening and integration of auditory, motor, predictive‐timing, and reward‐related systems (Figure 1). The hypothesis is organized around four core principles.

#### 2.1.1 Ancient, Conserved Architecture

The basic auditory cortical organization—including ventral and dorsal processing streams—is shared across primates, indicating that the neural substrate for auditory–motor interactions long predates humans (Figure 1C) [67].

#### 2.1.2 Gradual Strengthening of Dorsal Auditory–Motor Pathways

Human evolution primarily involved quantitative enhancement of conserved auditory–motor networks, including the dorsal auditory pathway and arcuate fasciculus, together with increasingly integrated cortico‐basal‐ganglia–thalamocortical timing circuits. These changes supported more precise sensorimotor integration, auditory working memory, vocal control, and predictive timing, rather than the emergence of entirely novel pathways (Figure 1A) [54, 55, 68].

#### 2.1.3 Increasing Integration of Reward Mechanisms

Reward‐related mechanisms, already present within the auditory cortex and involved in linking sounds, actions, and outcomes in nonhuman primates, became increasingly integrated with auditory–motor circuits during evolution. This integration reinforces temporal prediction, action selection, and learning, helping to motivate and stabilize rhythmic synchronization. Reward is, therefore, considered an essential evolutionary component of beat perception and synchronization rather than merely a consequence of musical experience (Figure 1B) [69].

#### 2.1.4 Progressive Coupling Rather Than Qualitative Innovation

Beat perception and synchronization evolved through progressively tighter bidirectional coupling among auditory prediction, motor planning, working memory, timing, and reward networks. Consequently, GAER predicts a continuum of rhythmic abilities across primates: component processes such as isochrony detection and tempo matching are broadly shared, whereas spontaneous, flexible beat synchronization requiring predictive control of both period and phase emerges only with increasing functional integration of these conserved neural systems (Figure 1A,B) [55, 60, 70].

## 3 Ontogeny

In developmental psychology and developmental neuroscience, comparisons between adults and infants highlight both continuity and transformation in neural and cognitive function. The nervous system in infancy is not a blank slate; rather, it reflects a combination of genetically guided developmental programs and early‐life experience [71]. Neurodevelopment follows highly conserved biological trajectories, wherein core structures and functional networks emerge according to endogenous timelines shaped by evolutionary constraints [72]. At the same time, experience‐dependent mechanisms—including synaptic pruning, myelination, and activity‐driven network specialization—progressively refine these neural systems [73]. This interaction between intrinsic developmental pathways and environmental input ensures that the infant brain begins life with considerable processing potential [74], while allowing maturation to track the organism's specific physical, social, and cultural environment.

Evidence from auditory neuroscience and developmental psycholinguistics indicates that infants possess a foundational capacity for processing complex acoustic information. Even in early infancy, neural responses reveal sensitivity to pitch contours, temporal regularities, and spectral structure [12]—acoustic dimensions that are integral to both music perception and language processing [75, 76]. These early competencies extend to the detection of rhythm, prosody, and phonemic patterns, supporting the notion that human infants are equipped with specialized auditory mechanisms that facilitate rapid learning in socially and biologically relevant domains [77, 78]. However, these abilities manifest at a coarse level relative to adults: while infants can discriminate changes in pitch or rhythm, their precision, consistency, and noise tolerance remain limited [79].

The developmental divergence between infant and adult auditory abilities can be traced to both perceptual exposure and embodied practice. Perceptual learning across childhood tunes auditory circuits to culturally specific pitch systems, linguistic categories, and rhythmic structures, narrowing broad early sensitivities into highly efficient, domain‐relevant representations [80, 81, 82]. Beyond passive exposure, active motor engagement plays a critical role in refining cognitive and perceptual skills [83, 84]. Speaking, singing, and playing musical instruments provide sensorimotor feedback loops that strengthen predictive timing [85], improve control of vocal and manual articulators, and enhance fine‐grained auditory discrimination. Thus, adult‐level refinement depends not only on accumulating sensory input but also on participating in structured actions that leverage and reinforce auditory−motor coupling [86].

This developmental trajectory underscores an important principle: infants already possess much of the architectural capacity required for sophisticated sound processing, yet their performance reflects a system still undergoing calibration and specialization [87]. The ontogeny of beat perception—embedded in this broader auditory and sensorimotor context—emerges from the same dual forces: genetically guided early readiness and gradual, experience‐driven optimization. Understanding how these forces interact provides a framework for interpreting similarities and differences in rhythmic perception across the lifespan, and for identifying which aspects of beat processing represent innate neurocognitive predispositions versus those shaped by cultural participation, motor practice, and exposure to structured sound.

### 3.1 Prenatal Foundations of Auditory and Rhythmic Processing

The onset of human sound experience is temporally constrained by the maturation of the auditory system: peripheral structures and central pathways support robust responsiveness by the early third trimester, as shown anatomically and by fetal magnetoencephalography recordings that detect auditory mismatch responses from approximately 28 weeks’ gestation [88, 89], with continuity of these responses observed after birth in preterm and term neonates. Within the uterus, the fetal auditory environment is dominated by low‐frequency sound energy, owing to the combined effects of sound transmission through maternal tissues and fluids and the maturation of the fetal auditory system [90]; and, as a result, higher‐frequency spectral detail is attenuated while the coarse temporal envelope, prosody, and rhythm of speech—and other slow periodicities—are preserved and dominate fetal input [91]. The most salient periodic sources include the maternal cardiovascular cycle, breathing, and movement [92], yielding millions of heartbeat cycles and motion‐related modulations before birth. These endogenous physiological rhythms, combined with external speech rhythms and environmental sonic patterns, provide a structured temporal scaffold that shapes early auditory encoding [93]. Comparative theoretical work even argues that key universals of musical rhythm may be partially rooted in this fetal rhythmic environment, wherein cyclic maternal physiology provides prototypical temporal patterns for later rhythmic perception [94]. After delivery, airborne, broadband sound becomes available, marking the transition into a conventional listening environment; however, this environmental shift overlays a nervous system already calibrated by prenatal rhythmic and prosodic exposure, as evidenced by experience‐dependent plasticity in preterm infants exposed to maternal voice and heartbeat [95].

Emerging neuroimaging evidence indicates that early musical exposure can shape functional organization in auditory and broader neural networks, but the current evidence should be interpreted cautiously. In preterm infants, music interventions in the neonatal intensive care environment have been associated with changes in resting‐state functional connectivity, including networks involved in salience processing and sensory integration, rather than providing direct evidence that newborns generally display coordinated auditory–limbic–motor responses to musical structure [96]. Related work suggests that familiar prenatal music can modulate newborn functional connectivity after birth, consistent with effects of prenatal auditory exposure on early brain organization [97]. However, these findings speak primarily to experience‐dependent plasticity and functional connectivity, not yet to beat perception or meter‐specific processing [87]. These findings align with ecological and dynamical systems accounts emphasizing that rhythmic perception and production capacities emerge through the embodied coupling of neural, physiological, and motor systems from the prenatal period onward [98]. Such frameworks resonate with proposals that the maturation of human rhythmic behavior is closely linked to locomotor development, with prenatal vestibular and proprioceptive exposure followed by postnatal bipedal motor experience supporting the progressive stabilization of beat‐related timing mechanisms [99]. Thus, prenatal auditory and sensorimotor rhythms not only prepare the auditory system for postnatal sound processing but may also act as foundational scaffolds for later musicality and rhythmic entrainment.

Evidence from fetal, premature, and newborn studies nevertheless suggests that sensitivity to temporal regularities is present very early in development. Recent fetal neurophysiology work indicates that late‐gestation fetuses encode auditory rhythmic structure, including responses to basic periodicity and more complex nested temporal organization [100]. In premature neonates, electroencephalography (EEG) studies show mismatch responses to rhythm violations at 30–34 weeks gestational age, suggesting that the immature cortex can detect deviations from recently established temporal regularities before term‐equivalent age [101, 102, 103]. These findings support the presence of early rhythm sensitivity, but they do not by themselves establish adult‐like beat or meter perception. In particular, violation responses may reflect several mechanisms, including stimulus‐driven tracking, local regularity learning, short‐horizon expectancy, or predictive processing over temporal structure.

At birth, EEG evidence shows that newborns respond differently to omissions depending on their metrical position in a rhythmic sequence. In the classic omission paradigm, omissions at the downbeat elicited mismatch responses even when the downbeat was not acoustically accented, suggesting sensitivity to temporal position within a repeating pattern [12, 13, 104]. More recent work has attempted to separate beat‐based timing from statistical order learning by comparing isochronous and jittered sequences; the presence of metrical‐position effects in isochronous but not temporally jittered sequences suggests that newborn responses cannot be explained by transitional probabilities alone [12, 13, 104]. This provides more direct support for early beat‐related timing mechanisms than studies of general musical or language processing alone. Still, the relevant conclusion should be framed narrowly: newborns show neural sensitivity to periodic temporal structure and to violations at metrically salient positions, not necessarily fully elaborated hierarchical meter perception.

The distinction between tracking and anticipation is critical. Neural tracking refers to phase‐locked or frequency‐specific responses aligned with the temporal structure of the stimulus. Anticipation or prediction requires stronger evidence, such as responses to omitted expected events, pre‐event neural dynamics, differential responses to violations when local acoustics are controlled, or model‐based evidence that a generative account with temporal predictions explains the data better than evoked‐response or adaptation‐based alternatives. MMN findings are compatible with predictive accounts because they often involve responses to unexpected or omitted events, but they can also reflect adaptation, auditory change detection, or learned local regularities. Similarly, frequency‐domain entrainment measures can index sensitivity to rhythmic periodicities, but spectral peaks alone do not uniquely demonstrate endogenous oscillatory entrainment or predictive meter inference.

Behavioral and motor evidence provides a complementary but still limited line of support. Newborns can modulate crawling‐related movements in response to native‐language speech rhythm, showing that auditory rhythm can influence neonatal motor output [104, 105]. This finding is relevant to embodied and sensorimotor accounts of rhythm development, but it should not be treated as direct evidence for musical beat perception. Rather, together with EEG studies of rhythm violation and omission responses, it suggests that auditory timing, motor regulation, and early temporal regularity processing are coupled from the beginning of postnatal life. The stronger claim, therefore, is not that infants are born with mature beat perception, but that they possess early beat‐relevant timing capacities that may scaffold later meter perception, sensorimotor synchronization, and musical entrainment.

### 3.2 Once More: Predictability Versus Statistical Learning Versus Beat Processing

A central conceptual and methodological challenge in early‐development rhythm research lies in distinguishing beat processing from statistical learning, given that the stimulus paradigms necessarily rely on highly controlled and often simplified rhythmic structures. Beat perception is typically defined as the extraction of an underlying periodic pulse that supports temporal prediction, whereas statistical learning involves tracking transitional probabilities or distributional regularities across events. In principle, the temporal dynamics of beat induction—requiring anticipation of future time points rather than detection of sequential contingencies—offer one route to differentiation. However, in practice, such dissociation is difficult to demonstrate in neonates and preterm infants, where both perceptual and neural measures are constrained and where rhythmic stimuli often contain co‐occurring temporal regularities and statistical patterns. While studies such as Háden et al. [13] argue that newborns’ responses cannot be fully explained by transition‐probability learning alone, current evidence does not conclusively separate beat‐based temporal prediction from statistical learning mechanisms, either in infancy or in more mature systems. Rather than treating the two as mutually exclusive, it may be more appropriate to consider beat perception and statistical learning as partially overlapping processes in early development, where temporal prediction mechanisms could emerge from, interact with, or scaffold upon more general learning capacities.

Methodologically, the field now needs designs that can distinguish among competing mechanisms. Useful innovations would include direct comparisons of isochronous and jittered controls, acoustic‐spectrum matched stimuli, omission paradigms that separate local expectancy from global metrical position, preregistered model comparisons between predictive, entrainment, adaptation, and evoked‐response accounts, and longitudinal designs linking fetal or neonatal neural responses to later rhythm perception and synchronization. Combining EEG or magnetoencephalography (MEG) with movement tracking, autonomic measures, and computational modeling would also help determine whether early neural responses reflect passive tracking, short‐term regularity learning, or genuine predictive timing.

### 3.3 Infancy (2–24 Months)

By the end of the first half‐year of life, infants demonstrate striking sensitivity to musical rhythm, including the ability to categorize rhythmic sequences according to meter and group auditory patterns based on underlying beat structure [77, 106]. Between 5 and 7 months, infants use metrical cues to organize both rhythms and melodies and begin to show selective preference for rhythms consistent with the metrical structures of their cultural environment, indicating early perceptual attunement and the onset of culturally shaped rhythmic learning [10, 14]. Neural evidence from frequency‐tagging paradigms demonstrates that infants entrain to beat‐ and meter‐related frequencies in music, with selective enhancement at metrical frequencies beyond low‐level acoustic cues [107, 108]. Similar entrainment signatures appear for complex musical rhythms, suggesting capacity for hierarchical temporal structure, and are observed across auditory modalities including live maternal singing [109, 110]. Moreover, neural phase‐tracking studies reveal that rhythmic alignment emerges early and may scaffold later music and language outcomes [103, 104, 111, 112]. These shifts reflect a transition from broad, universal sensitivity toward rhythm into more fine‐tuned beat processing mechanisms. Still, despite this progress, the infant system remains imprecise—hierarchical meter representation is emergent but not adult‐like, and motor synchronization remains rudimentary through the second year—indicating ongoing development of neural, perceptual, and motor integration [9].

### 3.4 Early Childhood (2–6 Years, Preschool)

During early childhood, beat perception and production abilities undergo significant refinement, yet both remain highly variable and context‐dependent. Behavioral studies consistently show that children aged 2−6 can detect and act on beat structure, but their synchronization is unstable and strongly dependent on salient temporal cues such as accents, tempo regularity, and interactive scaffolding. Spontaneous motor tempo studies reveal that very young children produce rhythmic movements naturally but struggle to align them precisely to external pacing until later preschool years; for instance, 2½‐ and 4‐year‐olds exhibit spontaneous rhythmic behaviors but show limited accuracy when attempting synchrony with an external beat [113]. Joint‐drumming paradigms reveal that children's synchronization improves when embedded in socially contingent contexts, suggesting that interpersonal interaction and shared intentionality facilitate temporal alignment [114, 115]. Methodologically, these paradigms typically use motion capture, tapping interfaces, or video‐coded body movements to index entrainment precision and phase correction strategies.

EEG and neural entrainment studies provide converging evidence that cortical beat tracking mechanisms are present but not fully mature in this age range. Preschoolers show neural responses to beat‐related frequencies in music, but with broader tuning and greater variability than adults [116]. Children with dyslexia exhibit weaker low‐frequency entrainment and reduced alignment between neural rhythms and tapping behavior, supporting links between auditory−motor coupling and language development [117]. Relatedly, electrophysiological work examining prediction mechanisms in rhythm tasks finds that 5‐ to 6‐year‐olds rely on beat‐based and non‐beat‐based timing strategies, with individual differences in predictive timing associated with broader cognitive abilities [118]. Notably, beat‐based timing remains more fragile than interval‐based timing at this stage, underscoring developmental transitions in the hierarchy of timing mechanisms. Performance remains heterogeneous across children, and sustained beat maintenance without external cueing is typically poor. Younger children rely on salient acoustic structure and social entrainment cues, and motor control limitations constrain synchronization accuracy.

### 3.5 Middle Childhood/Adolescence (7–14 Years)

Early research established clear developmental trends in children's rhythm and beat perception. Classic studies show that children's ability to maintain a steady beat improves with age and musical experience. Spontaneous tapping rates become slower and more consistent throughout childhood, with musically trained children demonstrating greater stability and more accurate synchronization than their untrained peers [119]. Children's synchronization performance varies with stimulus type: isochronous sequences tend to be easier for younger children, whereas successful synchronization with more complex rhythmic patterns typically emerges closer to age 10 [120]. Early findings also highlight that metric accents can support children's discrimination and production of rhythmic patterns, though producing accents reliably requires motor control that develops gradually between ages 6 and 8 [121]. Neural time‐keeping studies using EEG and MEG show that children's cortical entrainment to rhythmic stimuli strengthens throughout middle childhood, reflecting increasing precision in temporal prediction and beat tracking [121]. These studies demonstrate that 7‐ to 10‐year‐old children show reliable tracking of the beat level in music but less consistent tracking of higher‐level metrical structure (e.g., duple vs. triple meter), which continues to mature into early adolescence.

Motor synchronization research also reinforces the idea that temporal variability decreases with age. For example, longitudinal and cross‐sectional studies show that children steadily improve in tapping consistency, phase accuracy, and anticipatory timing between ages 6 and 12 [122, 123, 124]. Complex contexts—such as syncopation, tempo acceleration, or polymetric cues—pose challenges for younger children, who tend to fall back on simpler temporal regularities. As in earlier work in adults [125, 126, 127], musical training is consistently associated with more accurate beat alignment [11], greater temporal precision, and better adaptation to tempo changes [128, 129]. Recent developmental studies also highlight the role of sensorimotor entrainment: rhythm skills depend on the integration of auditory perception with coordinated movement. Research using drumming, stepping, and body‐percussion paradigms indicates that children more effectively internalize beat structure when allowed or encouraged to move, and that body‐based engagement enhances the accuracy of beat induction and metrical inference [130, 131]. These findings align with older work showing that response mode (clapping, chanting, stepping) influences accuracy depending on the child's age [131] and motor experience.

Overall, rhythm and beat perception undergo steady improvement through the school‐age years. Children gradually develop more stable internal timekeeping, finer sensitivity to metrical cues, and better synchronization accuracy—abilities influenced by maturation, experience, and opportunities for active rhythmic engagement.

### 3.6 Frequency Tagging

Frequency‐tagging EEG has become a central method for studying neural responses to musical rhythm in adults. By presenting isochronous or metrically structured auditory sequences and examining steady‐state responses at stimulus‐related frequencies, these studies often report spectral peaks at the beat and at metrically implied frequencies—even when those accents are not physically present in the acoustic signal [132, 133, 134]. Such findings have been interpreted as evidence that neural activity reflects not only bottom‐up tracking of acoustic periodicities but also top‐down metric interpretation, imagery, and attentional organization [135, 136]. Frequency‐tagging paradigms have, therefore, been widely used as markers of beat and meter perception and have also been extended to speech‐like and naturalistic rhythmic stimuli.

However, the interpretation of these responses remains actively debated. A recent registered multilaboratory replication study failed to reproduce one of the key findings of Nozaradan et al., namely, enhanced responses at meter‐related frequencies that were weak or absent in the stimulus spectrum itself [120]. In parallel, computational modeling work has shown that spectral peaks at beat frequencies are not uniquely diagnostic of endogenous neural entrainment, as both oscillatory entrainment models and models based on the superposition of successive evoked responses can account for measured EEG spectra and behavioral synchronization data [137, 138]. More broadly, current debates concern whether frequency‐tagged responses primarily reflect endogenous oscillatory alignment, evoked‐response accumulation, attentional selection, predictive inference, or interactions among these mechanisms. Although frequency‐tagging remains a powerful method for characterizing neural sensitivity to rhythmic structure, the extent to which specific spectral peaks uniquely index internally generated beat or meter representations remains unresolved.

Complementing these steady‐state approaches, studies of beta‐band (∼13–30 Hz) oscillations emphasize the predictive and sensorimotor aspects of rhythm processing. In adults, MEG studies show that beta power typically decreases following auditory events and rebounds prior to expected beats, a pattern often interpreted as reflecting predictive timing and auditory–motor coordination even in the absence of overt movement [139]. Theoretical and review work further links beta oscillations in cortico–basal ganglia networks to beat‐based timing and sensorimotor integration, proposing that beta activity helps maintain an internally generated temporal framework against which incoming rhythms are evaluated [140]. Yet, here too, interpretation remains debated. Beta dynamics may reflect temporal prediction, motor simulation, attentional allocation, maintenance of temporal context, or broader sensorimotor coordination processes rather than a dedicated beat‐specific mechanism. Thus, while findings from beta‐band studies and frequency‐tagging paradigms—including reports of neural responses to implied or “missing” beats—are broadly compatible with predictive and entrainment‐based accounts of rhythm perception, they do not yet uniquely discriminate among competing mechanistic models.

Similar interpretational challenges arise in MMN studies of rhythm and meter. MMN responses to omissions, syncopations, or metrically unexpected events are often taken as evidence that listeners generate predictions about future rhythmic structure, including hierarchical metrical expectations. However, it remains difficult to distinguish whether such responses reflect genuine predictive coding, adaptation effects, sensitivity to local statistical regularities, attentional orienting, or more general auditory change detection processes. This issue is particularly important in developmental work, where MMN responses in infants and newborns are frequently interpreted as evidence for early beat or meter perception despite ongoing debate about what level of temporal representation these paradigms actually index. Together, these debates highlight both the importance and the current limitations of MMN and frequency‐tagging methods: while they provide sensitive markers of neural sensitivity to rhythmic structure, the precise computational and neurophysiological mechanisms underlying these responses remain incompletely resolved.

Aging further illustrates the complexity of these interacting mechanisms. Older adults show reduced precision in neural responses to rhythm and meter and weaker encoding of metrical accents [140]. Aging also appears to alter the balance between bottom‐up [122] tracking of auditory rhythms and top‐down attentional modulation, potentially impairing the ability to maintain stable temporal predictions in complex auditory scenes [141]. At the same time, rhythmic musical engagement may partially counteract these declines by strengthening connectivity among networks involved in timing, attention, and executive function [142]. Together, these findings suggest that neural responses to rhythm emerge from interacting sensory, predictive, attentional, and motor processes whose relative contributions may shift across the lifespan.

### 3.7 Integration Using Comparable Paradigms

Comparing abilities across developmental stages and species is crucial because it lets us tease apart what is uniquely human, what is shared with other animals, and what emerges early versus late in life. This approach was successfully used for nonadjacent sequence learning [143, 144, 145]. Developmental data show which aspects of rhythm and melody perception are present in newborns and infants, revealing biologically grounded predispositions that are in place before extensive postnatal learning and cultural shaping [78] (but see prenatal experiences above). Phylogenetic comparisons, in turn, identify which components of musicality—such as beat‐based timing or complex rhythmic prediction—are conserved across species and which appear to be evolutionary specializations of our lineage [21, 146]. Within a biomusicological framework, integrating ontogenetic and phylogenetic evidence addresses Tinbergen's classic questions about mechanism, ontogeny, phylogeny, and function, and provides powerful constraints on theories of how neural timing and predictive processing give rise to specifically human forms of musicality [40, 147].

Using closely matched auditory oddball paradigms, Ladinig et al. [148], Winkler et al. [12], and Honing et al. [45] systematically probed rhythm and meter processing in human adults, human newborns, and rhesus monkeys (respectively) with variants of the same metrical rhythmic pattern. Across all three studies, MMN or MMN‐like responses were used to index whether listeners formed predictive representations of temporal structure. In adults, metrically strong positions elicited enhanced behavioral and neural responses compared with weak positions, even under passive listening, indicating preattentive hierarchical meter processing [149]. However, there were concerns that the ability of the listener to recognize longer patterns and the acoustic context of an omission can influence the ERP response to sound omissions in a rhythm [150] that were addressed in later paradigms [37] (see next paragraph). Newborns tested during natural sleep showed MMN responses to downbeat omissions only in a metrical context, suggesting that a periodic pulse can be extracted from complex rhythms very early in life [12]. In contrast, rhesus monkeys displayed MMN‐like responses to simple pitch and timing deviants, but failed to differentiate between downbeat and nondownbeat omissions, indicating sensitivity to local rhythmic grouping without evidence for an underlying beat and supporting the view that human‐like beat induction and hierarchical metrical processing may be uniquely human specializations [53].

Bouwer et al. [37] directly tested whether neural responses previously attributed to beat perception could be reduced to simpler sequential or statistical learning by orthogonally manipulating metrical structure and transition probabilities in rhythmic patterns, showing that MMN responses to beat‐related omissions persisted even when sequential predictability was minimized and that attention and musical abilities affected only later ERP components. Honing et al. [45] extended this logic to rhesus monkeys using stimuli that separated interval‐based timing from metric structure, finding that monkeys reliably detected violations of isochrony but still showed no evidence of beat‐based prediction, thereby strengthening the GAE hypothesis by demonstrating preserved interval timing but absent human‐like beat induction. Finally, Háden et al. [13] applied a similar dissociation of metrical salience and transitional probabilities in newborns and found that enhanced responses to beat‐related omissions remained even when statistical regularities did not support those expectations, indicating that infant beat processing cannot be fully explained by low‐level statistical learning and instead reflects an early sensitivity to hierarchical temporal structure.

Di Liberto et al. [150] used intracranial and scalp EEG combined with a computational model of melodic expectation to show that human superior temporal regions encode both the expectedness of upcoming notes and the magnitude and timing of melodic prediction errors during naturalistic music listening, revealing rapid (tens to hundreds of milliseconds) cortical dynamics for melodic prediction. Bianco et al. [64] applied a similar modeling framework to macaques and found temporally precise neural responses to note‐level deviations that were tightly coupled to local acoustic changes, indicating that nonhuman primates primarily generate short‐range auditory expectations rather than the longer‐range hierarchical encoding observed in humans. Extending this line of work developmentally, Bianco et al. [158] showed that newborn humans produce early prediction‐related responses for rhythmic, but not melodic, violations, suggesting that while infants can track periodic timing and detect rapid rhythmic deviations, the longer‐range temporal integration required for melodic prediction emerges later in development. While these studies do not directly test beat perception, they point to a temporal gradient across species and development in which rapid, short‐timescale prediction is broadly conserved, whereas long‐range melodic prediction is only elaborated in the mature human brain.

Taken together, the three‐way comparisons across adults, newborns, and nonhuman primates offer a powerful framework for disentangling the phylogenetic and ontogenetic foundations of beat perception. By applying closely matched paradigms across species and developmental stages, these studies reveal which aspects of temporal prediction reflect ancient, evolutionarily conserved mechanisms and which constitute uniquely human specializations. The presence of beat‐sensitive neural responses in adults and newborns, contrasted with their absence in monkeys despite intact duration‐based timing, demonstrates that beat induction does not emerge gradually through learning or environmental exposure but is rooted in a species‐specific predisposition. At the same time, preserved interval timing across humans and monkeys highlights shared auditory temporal processing capacities inherited from a common primate ancestor.

These cross‐level comparisons also clarify the developmental trajectory of human temporal cognition. Newborn findings show that the neural infrastructure for rhythm‐based prediction is available from birth, ruling out explanations based solely on statistical learning or musical enculturation. Adult studies then reveal how this early‐developing beat mechanism becomes embedded within a richer hierarchical meter system through maturation and experience. In contrast, the consistent failure of nonhuman primates to generate beat‐based predictions—even when they succeed with isochrony or local temporal violations—underscores what is absent in primate development and evolution, helping isolate the neural computations that are uniquely elaborated in humans.

By integrating these ontogenetic and phylogenetic perspectives, three‐way comparisons make it possible to map the architecture of beat perception with far greater precision than single‐species or single‐age studies allow. They enable researchers to separate universal auditory timing mechanisms from those that emerged specifically within the human lineage, and to distinguish innate temporal prediction from abilities shaped by learning and cultural exposure. This comparative approach thus provides a rigorous empirical foundation for theories of musical cognition as an evolved, developmentally organized, and biologically constrained capacity.

## 4 Conclusion

In short, the comparative research spanning adults, newborns, and nonhuman primates has provided an exceptionally productive framework for identifying which components of beat perception are innate, which are acquired, and which are uniquely human. Yet, these advances also highlight a critical limitation of the field: most existing paradigms have focused on a relatively narrow set of electrophysiological markers, especially oddball‐evoked mismatch responses. To fully characterize the temporal and hierarchical dynamics of beat perception across species and development, future work will require diversifying the methodological toolbox, that is, to include a broader range of behavioral, neurophysiological, genetic, and modeling approaches that can better dissociate stimulus tracking from endogenous beat‐based prediction across ages and species [52, 151, 152, 153].

## Author Contributions

Writing and editing: H.H. and G.H.

## Conflicts of Interest

The authors declare that they have no conflicts of interest.
